# Early Reperfusion Following Ischemic Stroke Provides Beneficial Effects, Even After Lethal Ischemia with Mature Neural Cell Death

**DOI:** 10.3390/cells9061374

**Published:** 2020-06-01

**Authors:** Yasue Tanaka, Nami Nakagomi, Nobutaka Doe, Akiko Nakano-Doi, Toshinori Sawano, Toshinori Takagi, Tomohiro Matsuyama, Shinichi Yoshimura, Takayuki Nakagomi

**Affiliations:** 1 Department of Neurosurgery, Hyogo College of Medicine, 1-1 Mukogawacho, Nishinomiya, Hyogo 663-8501, Japan; yasuet@hyo-med.ac.jp (Y.T.); to-takagi@hyo-med.ac.jp (T.T.); 2 Department of Surgical Pathology, Hyogo College of Medicine, 1-1 Mukogawacho, Nishinomiya, Hyogo 663-8501, Japan; na-nakagomi@hyo-med.ac.jp; 3 General Education Center, Hyogo University of Health Sciences, 1-3-6 Minatojima, Chuo-ku, Kobe, Hyogo 650-8530, Japan; doe@huhs.ac.jp; 4 Institute for Advanced Medical Sciences, Hyogo College of Medicine, 1-1 Mukogawacho, Nishinomiya, Hyogo 663-8501, Japan; nakano@hyo-med.ac.jp; 5 Department of Therapeutic Progress in Brain Diseases, Hyogo College of Medicine, 1-1 Mukogawacho, Nishinomiya, Hyogo 663-8501, Japan; tomohiro@hyo-med.ac.jp; 6 Department of Biomedical Sciences, Ritsumeikan University, 1-1-1 Nojihigashi, Kusatsu, Shiga 525-8577, Japan; t.sawano1988@gmail.com

**Keywords:** ischemic stroke, early reperfusion, transient middle cerebral artery occlusion, permanent middle cerebral artery occlusion, central nervous system

## Abstract

Ischemic stroke is a critical disease caused by cerebral artery occlusion in the central nervous system (CNS). Recent therapeutic advances, such as neuroendovascular intervention and thrombolytic therapy, have allowed recanalization of occluded brain arteries in an increasing number of stroke patients. Although previous studies have focused on rescuing neural cells that still survive despite decreased blood flow, expanding the therapeutic time window may allow more patients to undergo reperfusion in the near future, even after lethal ischemia, which is characterized by death of mature neural cells, such as neurons and glia. However, it remains unclear whether early reperfusion following lethal ischemia results in positive outcomes. The present study used two ischemic mouse models—90-min transient middle cerebral artery occlusion (t-MCAO) paired with reperfusion to induce lethal ischemia and permanent middle cerebral artery occlusion (p-MCAO)—to investigate the effect of early reperfusion up to 8 w following MCAO. Although early reperfusion following 90-min t-MCAO did not rescue mature neural cells, it preserved the vascular cells within the ischemic areas at 1 d following 90-min t-MCAO compared to that following p-MCAO. In addition, early reperfusion facilitated the healing processes, including not only vascular but also neural repair, during acute and chronic periods and improved recovery. Furthermore, compared with p-MCAO, early reperfusion after t-MCAO prevented behavioral symptoms of neurological deficits without increasing negative complications, including hemorrhagic transformation and mortality. These results indicate that early reperfusion provides beneficial effects presumably via cytoprotective and regenerative mechanisms in the CNS, suggesting that it may be useful for stroke patients that experienced lethal ischemia.

## 1. Introduction

Ischemic stroke is a leading cause of death worldwide. Patients who experienced ischemic stroke were previously limited to treatment options, such as antiplatelet therapy and rehabilitation. However, recent therapeutic advances in reperfusion therapies, such as intravenous (IV) administration of recombinant tissue plasminogen activator (t-PA) and neuroendovascular treatment (e.g., mechanical thrombectomy) [1,2,3], have allowed some stroke patients to recover without sequelae. Recently, the therapeutic time window of IV t-PA was expanded up to 4.5 h [4] and has been indicated for use in stroke patients of unknown onset [5]. Mechanical thrombectomy has a larger therapeutic time window—up to 24 h when a mismatch between the ischemic core and hypoperfusion area is observed in a stroke patient’s imaging results [6,7]. These expanded indication windows will allow more stroke patients to benefit from treatment. However, several factors still limit application of these therapies, including the occluded brain artery location and limited therapeutic time windows. This is highlighted by mechanical thrombectomy, where only 13–20% of stroke patients are potentially eligible for treatment [7,8].

Although an increased number of stroke patients may receive these reperfusion therapies, it is necessary to consider additional indications that allow more stroke patients to receive treatment. Currently, if stroke patients experience severe ischemia diagnosed by computed tomography, magnetic resonance imaging (MRI), or other imagining, they are typically excluded from reperfusion therapies. However, in addition to neural cells (neurons and glia), brain cells include vascular cells (endothelial cells and pericytes), which have been found to be more resistant to ischemia/hypoxia [9,10,11,12,13,14]. Thus, it is possible that vascular cell-mediated healing processes may still function in ischemic areas even after lethal ischemia with the death of mature neural cells [14]. In support of this idea, a recent study using a mouse model of ischemic stroke indicated that early reperfusion, which was performed within 240 min following ischemic insult, had more beneficial effects than simply rescuing neural cells [11]. This suggests that early reperfusion may be an additional option for stroke patients, even if the ischemic areas have already undergone lethal ischemia with mature neural cell death. However, the mechanism of these beneficial effects and the long-term consequences of early reperfusion following lethal ischemia remain unknown.

The present study sought to clarify these issues using two types of mouse ischemic models. One was a 90-min transient middle cerebral artery occlusion (t-MCAO) that induces lethal ischemia; the other was permanent middle cerebral artery occlusion (p-MCAO). We evaluated the effects of early reperfusion following lethal ischemia at multiple time points (1, 3, 7, 28, and 56 d post stroke), including acute (1–7 d post stroke) and chronic periods (more than 21 d post stroke), as defined by a previous study [15], with a focus on histology and brain functions. Moreover, 2,3,5-triphenylteterazolium (TTC) staining 1 d post stroke showed that early reperfusion following 90-min t-MCAO was sufficient to cause lethal ischemia. Although immunohistochemistry confirmed that early reperfusion following 90-min t-MCAO did not rescue mature neural cells, including neurons and glia, it preserved the vascular cells within the ischemic areas at 1 d following 90-min t-MCAO compared to that after p-MCAO. Histology studies revealed that reperfusion in the t-MCAO model reduced the ischemic area size at 7 d post stroke, indicating that early reperfusion promoted the healing processes. Early reperfusion also promoted long-term healing, which was revealed by immunohistochemistry in term of increased neurons, astrocytes, oligodendrocytes, and vascular cells at 56 d following t-MCAO compared to that following p-MCAO. This enhanced recovery was also related to behavioral outcomes. Mice exposed to reperfusion in the t-MCAO model showed not only fewer functional deficits but also enhanced recovery of neurological deficits compared to those in the p-MCAO model at 1–56 d post stroke. Most importantly, we found that reperfusion did not increase instances of mortality or hemorrhagic transformation, and it reduced the blood–brain barrier (BBB) permeability, indicating its safety. These results prove early reperfusion to be an efficient and safe intervention following lethal ischemia, potentially due to its ability to promote vascular cell-mediated healing processes.

## 2. Material and Methods

### 2.1. Animals

Studies were completed in adult male CB-17/Icr-+/+Jcl (CB-17) mice aged six to twelve weeks (Clea Japan, Inc., Tokyo, Japan). Animals had access to food ad libitum. All procedures were performed in accordance with a protocol approved by the Animal Care Committee of Hyogo College of Medicine (License number: 17-051, 2019-10-2). Efforts were made to minimize the numbers of animals used and their suffering. Quantitative analyses were conducted by investigators who were blinded to the experimental protocol and sample identity.

### 2.2. Cerebral Ischemia Induction

Focal t-MCAO or p-MCAO was induced as previously described [10,12,13,14,16]. In brief, general anesthesia was induced by inhalation of 2% isoflurane, followed by maintenance of anesthesia by inhalation of 1.5% isoflurane. Rectal temperature was maintained at 35–37 °C using a heating lamp (Nipponkoden, Tokyo, Japan). Mice were placed in a lateral position, and a skin incision was made at the midpoint between the left orbit and the external auditory canal. Under an operating microscope (OMS-90; TOPCON CORPORATION, Tokyo, Japan), the upper part of the temporalis muscle was pushed aside to allow visual development for craniotomy and exposure of the left middle cerebral artery (MCA). A 1- to 2-mm burr hole was made against the skull using a dental drill (H021 Minimo; MINITOR CO., LTD, Tokyo, Japan) and the dura mater was opened and retracted carefully so as not to damage the surface of the brain.

For t-MCAO, the left MCA was transiently occluded using a 2 mm long 7-0 monofilament nylon suture (Tyco, Princeton, NJ, USA) distal to the olfactory tract crossing. The suture was rotated 180° clockwise to align horizontally with the artery for complete interruption of blood flow. Mice were returned to their cages controlled at 35–37 °C and kept without anesthesia until reperfusion for 20, 60, or 90 min. Mice were then placed under anesthesia and cerebral blood flow was restored by returning the nylon suture to its original position by counterclockwise rotation. The success of reperfusion by this procedure was confirmed by the detection of cerebral blood flow in the left MCA regions using laser speckle flowmetry (Omegazone laser speckle blood flow imager, Omegawave, Inc., Tokyo, Japan), as described [10]. In another set of experiments, p-MCAO was performed by electrocoagulation and disconnection of the distal portion of left MCA. Sham operations were performed following the same procedure, but the surgery was stopped after the dura mater was opened.

### 2.3. Infarct Volume Evaluation

Infarct volume was analyzed after 20-, 60, or 90-min t-MCAO and p-MCAO as previously described [14]. In brief, 1 d (24 h) after t-MCAO or p-MCAO, mice were deeply anesthetized with sodium pentobarbital (50 mg/kg). Brains were removed and coronal brain sections (2 mm thickness) were stained with 1% TTC (Sigma-Aldrich, St. Louis, MO, USA) for 20 min at 37 °C in the dark. Then, they were fixed in 4% paraformaldehyde (PFA)/phosphate-buffered saline (pH 7.4) to evaluate brain tissue viability. Images were captured using a microscopic digital camera system (Olympus, Tokyo, Japan). The TTC-unstained area of each slice was evaluated using National Institutes of Health Image (Image J) software (Version 1.62) as described [9,10,17,18]. The TTC-unstained volume was calculated by multiplying the area by thickness. Infarct volume was calculated as the sum of the value for each slice.

### 2.4. Preparation of Brain Samples Following Ischemia

Mice were treated with an intraperitoneal administration of sodium pentobarbital (50 mg/kg) and transcardially perfused with 4% PFA as described [10,12,13,14] 1, 3, 7, 28, or 56 d after ischemia induction. Whole brains were removed and subjected to post-fixation with 4% PFA for 24 h. They were then embedded in paraffin and sliced into 8 μm sections. Continuous sections from the center of the forebrain were subjected to hematoxylin and eosin (H&E) staining and immunohistochemistry. In another experiment, fixed brains were cryoprotected in 30% sucrose, frozen at −80 °C, and cut into 16-μm sections using a cryostat for double immunohistochemistry.

### 2.5. Histological Evaluation

Using the H&E-stained sections obtained from the same region of the brain for each animal, the size of the “ischemic areas” defined as the area within the border of the post-stroke area was analyzed using Image J software, as described [9,10,17,18]. The percent ischemic area was calculated according to Equation (1) [14,19]:

(1) $$\frac{Contralateral\;Hemispere\;Area-Intact\;Area\;of\;Infracted\;Hemisphere}{Contralateral\;Hemisphere\;Area\times 2}\times 100$$

In addition, to evaluate the inflammatory changes following ischemic stroke, the numbers of inflammatory cells morphologically identified as neutrophils or macrophages/microglia were measured within the ischemic areas [a total of 27 data points (9 points/section, 1 section/brain, taken from 3 brains)] using coronal brain sections obtained from the same region.

### 2.6. Immunohistochemistry

Following deparaffinization, sections (8 μm thickness) were subjected to heat treatment using a microwave for epitope retrieval in citrate buffer solution (pH 6.0; Abcam, Cambridge, UK) for 10 min. Then, the samples were incubated with primary antibodies against cluster of differentiation 206 (CD206) (1:50; mouse; R&D systems, Minneapolis, MN, USA), platelet derived growth factor receptor-beta (PDGFRβ) (1:200, goat, R&D systems), ETS-related gene (ERG) (1:200, rabbit, Abcam), nestin (1:100; mouse; Millipore, Temecula, CA, USA), microtubule-associated protein 2 (MAP2) (1:500, Sigma-Aldrich), S100β (1:2000, mouse, Abcam), neurofilament (1:1000, rabbit, Abcam), and CNPase (1:1000, Abcam) at 4 °C for 24 h. Primary antibodies reactions were completed using secondary antibodies harboring universal immunoperoxidase (*N*-Histofine Simple Stain Mouse MAX PO, Nichirei Corporation, Tokyo, Japan). Sections were stained with 3,3′-diaminobenzide tetrahydrochloride (Vector Laboratories Inc., Burlingame, CA, USA), followed by counterstaining with hematoxylin. Images were captured using a microscope (Olympus). CD206^+^, PDGFRβ^+^, nestin^+^, MAP2^+^, S100β^+^, neurofilament^+^, and CNPase^+^ cells or the number of ERG^+^ vessels were identified within the region of interest. Using coronal brain sections obtained from the same region for each animal, a total of 27 data points (3 points/section, 3 sections/brain, taken from 3 brains) were analyzed using Image J software and subjected to semi-quantitative analysis as previously described [9,10,17,18]. In this study, we defined the “ischemic area” as the area within the border of the post-stroke area and “peri-ischemic area” as the area within 100 μm from the border of the post-stroke area as described [10].

In another set of experiments, brain sections (16 μm thickness) were subjected to double immunohistochemistry using primary antibodies against nestin (1:100, mouse, Millipore), CD31 (1:100; rat; BD Pharmingen, San Diego, CA, USA), and PDGFRβ (1:500, goat, R&D systems), followed by incubation using Alexa Fluor 488- or 555-conjugated secondary antibodies (1:500; Molecular Probes, Eugene, OR, USA). Cell nuclei were stained with 4′,6-diamidino-2-phenylindole (DAPI; 1:500; Kirkegaard & Perry Laboratories, Inc., Gaithersburg, MD, USA). Images were captured using a confocal laser microscope (LSM780; Carl Zeiss AG, Oberkochen, Germany).

### 2.7. Cell Cultures

To investigate whether neural stem/progenitor cells (NSPCs) are present within the ischemic areas, ischemic tissues were carefully removed from the MCA 7 d following t-MCAO and p-MCAO as previously described [10,12,13,14]. In brief, the removed tissues were mechanically dissociated by passage through 18-, 23-, and 27-gauge needles to prepare a single-cell suspension. The resulting suspensions were incubated in Dulbecco’s Modified Eagle Medium/F-12 (Thermo Fisher Scientific, Rochester, NY, USA) containing 20 ng/mL of basic fibroblast growth factor (bFGF; PeproTech, Rocky Hill, NJ, USA), 20 ng/mL of epidermal growth factor (PeproTech), and 1% N_2_ supplement (Invitrogen, Carlsbad, CA, USA), and penicillin (100 U/mL). Neurosphere-like cell clusters were subjected to reverse transcription polymerase chain reaction (RT-PCR) 21 d after incubation (see below). Clusters were differentiated on poly-l-lysine-coated (0.05%) glass coverslips for 7 d in neurobasal medium (Invitrogen) containing bFGF, B-27 supplement (Invitrogen), and 2% fetal bovine serum as previously described [20]. The differentiated cell clusters were subjected to RT-PCR and immunohistochemistry using antibodies against Tuj1 (1:1000; mouse; Stemcell Technologies, Vancouver, BC, Canada), MAP-2 (1:1000, rabbit, Millipore), glial fibrillary acidic protein (GFAP) (1:1000, rabbit, Abcam), and myelin basic protein (MBP) (1:100, R&D systems), followed by Alexa Fluor 488- or 555-conjugated secondary antibodies (1:500; Molecular Probes).

### 2.8. Western Blot Analysis

Western blot analysis was performed as previously described [9,10]. In brief, mice were sacrificed 1, 3, or 7 d following 90-min t-MCAO and p-MCAO. Brain samples (10 μg) were obtained from the ipsilateral ischemic ipsilateral or the contralateral nonischemic contralateral MCA. They were then separated by sodium dodecyl sulfate-polyacrylamide gel electrophoresis. Separated proteins were electrophoretically transferred onto PVDF membranes (Bio-Rad, Hercules, CA, USA). Membranes were incubated with a primary antibody against albumin (1:2000, A90-134A, Bethyl Laboratories), followed by incubation with peroxidase-labeled secondary antibody (1:1000, 61-1620, Invitrogen). Antibody labeling of protein bands was detected with enhanced chemiluminescence reagents (Chemi-Lumi One, Nacalai, Tesque, Kyoto, Japan) according to the manufacturer’s instructions. Albumin levels were normalized to β-actin using Image J analysis software as previously described [10,12].

### 2.9. Reverse Transcription Polymerase Chain Reaction

Total RNA was extracted using an RNeasy Micro Kit (Qiagen, Hilden, Germany) as previously described [12,20,21,22]. In brief, total RNA was extracted from the differentiated cell clusters obtained after 90-min t-MCAO and p-MCAO. cDNA was amplified according to the manufacturer’s protocol. The primer sequences used in this study are listed in Table 1.

### 2.10. Behavioral Tests

Phenotypic behavioral differences were assessed in sham-operation, 90-min t-MCAO, and p-MCAO mice in accordance with previous reports [23,24,25,26,27], with minor modifications. The spontaneous activity, wire hang, and basket tests were performed at 1, 7, 28, and 56 d post stroke. Open-field, hot plate, Y-maze, passive avoidance learning, open space swimming, and forced swimming tasks were performed at 56 d post stroke.

#### 2.10.1. Spontaneous Activity Test

Spontaneous locomotor activity was measured by an animal activity monitoring apparatus (SUPERMEX, CompACT AMS ver. 3, Muromachi Kikai Co., Tokyo, Japan). Mice were individually placed in plastic cages (20 × 25 × 15 cm) containing fresh sawdust. They were then put in an isolation chamber (45 × 45 × 45 cm) equipped a ceiling sensor for 30 min. The system monitors mouse movement by measuring changes in heat energy in the covered field.

#### 2.10.2. Wire Hang Test

The wire hang test was conducted using a square wire mesh plate (30 × 30 cm) made of warp and weft wires (0.8 mm in diameter) loosely woven in a mesh shape. Each mouse was placed on the wire mesh plate and allowed to acclimate to the environment for 10 s. The testing plate was then gently inverted and secured to the top of a cubic open-topped glass box (25 × 25 × 25 cm). The latency to fall was measured, with a maximum trial time of 180 s.

#### 2.10.3. Basket Test

The basket test was conducted using a cuboid wire mesh basket (30 × 30 × 40 cm). Each mouse was placed on the bottom surface of the basket and allowed to acclimate to the environment for 10 s. The basket was then gently inverted and put on a flat plate (40 × 35 × 5 cm) where sawdust was spread. The latency to climb down the vertical walls of the basket to the flat plate was recorded, with a maximum trial time of 180 s. Three trials were completed for each mouse with an interval of 10 min.

#### 2.10.4. Open Field Test

A cubic open-field box made of transparent acrylic plates without a lid (30 × 30 × 30 cm) was housed in a ventilated soundproof chamber (Taiyo Electric Co. Ltd., Osaka, Japan). An overhead incandescent light bulb provided room lighting that measured approximately 110 lux at the center of the test arena. A fan attached to the upper part of the wall at one end of the chamber generated white noise (45 dB) inside the chamber. Two infrared beams were attached 2 cm above the floor at a 10 cm distance on each wall of the box. A flip-flop circuit was set up between the two beams. The total number of circuit breaks was used to quantify locomotive behavior. Each mouse could freely explore the open-field arena for 10 min on 2 consecutive days.

#### 2.10.5. Hot Plate Test

The hot plate test was conducted using a thermo-controllable aluminum plate (Model MK-350B, Muromachi Kikai Co., Tokyo, Japan). The mice were ran through eight test trials at with an interval of 10 min. The temperature of the surface of the aluminum plate started at 50 °C and was raised in 2 °C intervals to 64 °C over the trials. Each trial took 20 s, and the latency to jump or lick paw was recorded.

#### 2.10.6. Y-Maze Task

The Y-maze task was performed in a Y-shaped maze with identical arms (length: 40 cm, width: 3 cm, height: 20 cm). The arms were labeled A, B, or C and diverged at 120° angles from a central area. The apparatus was set on a pedestal (height: 30 cm) that was enclosed by four white walls (height: 120 cm). The apparatus was illuminated by indirect lighting that was 150 lux at the maze floor. Each mouse was placed in the central area and allowed to freely explore the maze for 5 min. A mouse was considered to have entered an arm when all 4 paws were positioned in the arm runway. An alternation was defined as entry into all 3 arms on consecutive occasions (ABC, CBA, or BAC). The maximum alternation was calculated as the total number of arm entries minus 2, and the percentage of alternation was calculated as Equation (2):

(2) $$\frac{Actual\;Alteration}{Maximum\;Alteration}\times 100$$

#### 2.10.7. Passive Avoidance Learning

All mice were trained in a step-through-type passive avoidance apparatus consisting of a light and a dark compartment (15 × 15 × 15 cm each) with a grid floor. A guillotine door separated the two compartments. In the conditioning trial, mice were individually placed in the light compartment for 10 s, and then the door was opened. Once the mouse moved into the dark compartment, the guillotine door was closed, and 10 s later, a scrambled electrical shock (160 V, 5 s) was delivered through the grid floor. The retention test trial without any shock was conducted 24 h later. Each mouse was placed in the light compartment and the latency to enter the dark compartment was measured with a maximum limit of 180 s.

#### 2.10.8. Open Space Swimming Test

The open space swimming test was conducted using a circular pool (inside diameter: 95 cm, depth: 35 cm) enclosed by white featureless walls (width: 130 cm, height: 120 cm). The pool was filled with water to a 20-cm depth and maintained at 22 ± 1 °C. Titanium oxide was added to make the water opaque. Each mouse was placed with its head facing the outer edge of the pool and allowed to swim or float freely for 10 min. All trials were recorded with a digital video camera placed above the maze. The total amount of immobility was calculated by measuring the duration of time (s) that the mouse traveled below the specified threshold velocity of 3 cm/s. This threshold was validated by comparing the manually rated immobility scores with scores from the computerized video-based tracking system in preliminary studies.

#### 2.10.9. Forced Swimming Test

The forced swimming test was conducted using a small cylindrical tank (inside diameter: 18 cm, depth: 29 cm) containing water to a 15-cm depth maintained at 24 ± 1 °C. The tank was housed in a ventilated soundproof chamber (45 × 45 × 45 cm). A fan attached to the upper part of the wall at one end of the chamber generated white noise (45–50 dB) inside the chamber. An incandescent bulb attached to the chamber lid provided indirect lighting that measured approximately 150 lux at the water surface. The chamber was equipped with the SUPERMEX system (CompACT FSS ver. 2, Muromachi Kikai Co., Tokyo, Japan). Mice were individually placed in the tank for 6 min and the software calculated the duration of immobility by summing the time segments (s) where the magnitude of the mouse’s movements was below a pre-set threshold.

### 2.11. Statistical Analysis

Data are presented as mean ± standard deviations. Statistical comparisons between multiple groups or samples were determined using one-way analysis of variance (ANOVA), followed by multiple comparison. Where indicated, comparisons between the stroke groups (90-min t-MCAO vs. p-MCAO) were performed using Student’s *t*-test. The incidence of hemorrhagic transformation was analyzed using a chi-squared test. The survival rate was analyzed using a logrank test. Behavioral tests were analyzed using one-way ANOVA, followed by multiple comparison or a repeated measure ANOVA with treatment group (sham-operation, 90-min t-MCAO, or p-MCAO) as the between-subject factor and repeated measures (session, trial, or time) as the within-subject factor as previously described [23,25]. *p*-values <0.05 were considered statistically significant.

## 3. Results

### 3.1. Evaluating the MCAO Time Course that Produces Lethal Ischemia

**Figure 1:**
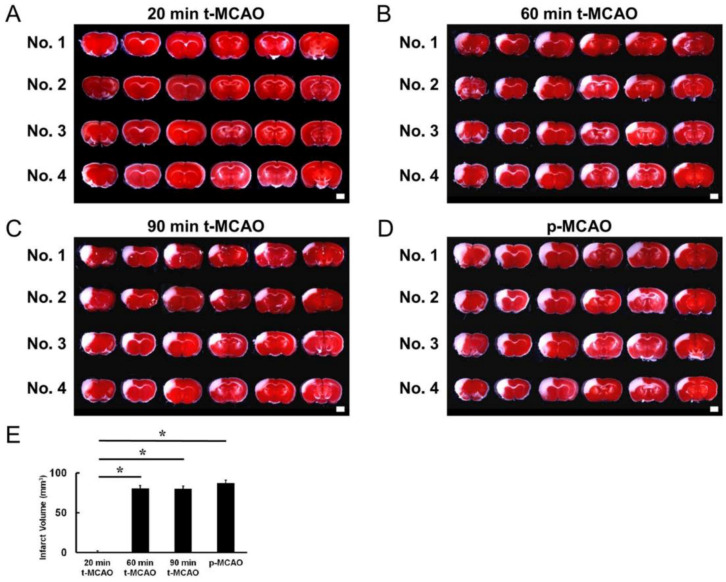
TTC staining at 1 d post stroke in four mice per condition (No. 1, 2, 3, 4). Following 20-min t-MCAO, partial ischemia was observed in 1/4 mice (No. 4) (**A**). In contrast, ischemia was observed in all mice after 60-min t-MCAO (**B**), 90-min t-MCAO (**C**), and p-MCAO (**D**). Compared to 20-min t-MCAO, infarct volume was significantly higher in mice after 60-min t-MCAO, 90-min t-MCAO, and p-MCAO, but not different between these groups (**E**). Scale bars = 2 mm (**A**–**D**). * *p* < 0.05 vs. 20 min t-MCAO (**E**) (*n* = 4, for each group). Abbreviations: p-MCAO, permanent middle artery occlusion; t-MCAO, transient middle cerebral artery occlusion.

The MCAO duration that induces lethal ischemia was observed using t-MCAO and p-MCAO—highly reproducible mouse model of stroke [11,14,16]—over various durations. Brain sections that were stained with TTC 1 d post stroke showed that t-MCAO with reperfusion at 20-min produced lethal ischemic injury in 25% of mice, indicating that it was insufficient to induce lethal ischemic injury (Figure 1A). However, TTC staining showed that lethal ischemic injury was induced in all mice following t-MCAO with reperfusion at 60-min (Figure 1B) or 90-min (Figure 1C) and p-MCAO (Figure 1D). Compared with mice that received reperfusion at 20-min, the stroke volume was significantly greater in mice exposed to 60- and 90-min t-MCAO and p-MCAO. However, there was no significant difference between these three groups (Figure 1E).

### 3.2. Early Reperfusion Accelerates Reductions in Ischemic Area Size

Our data indicated that 60-min t-MCAO sufficiently induced lethal ischemic injury in this mouse strain. To investigate the effect of early reperfusion after lethal ischemia, we compared brain histology at 1 d post stroke in mice exposed to 90-min t-MCAO and p-MCAO.

**Figure 2:**
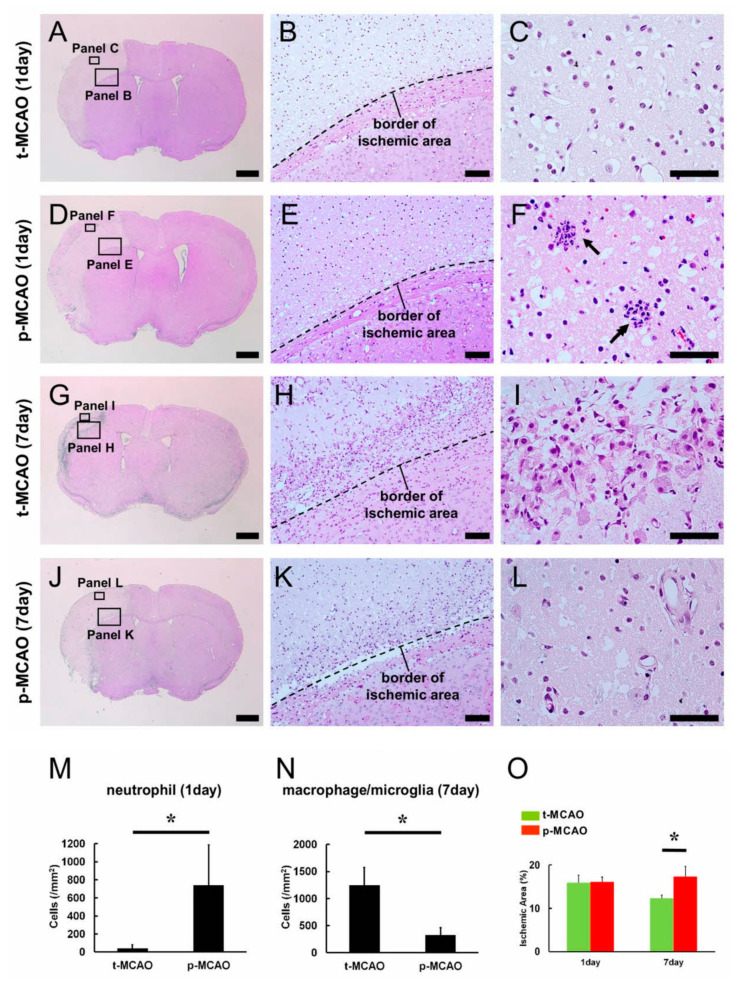
H&E staining of brain sections obtained following 90-min t-MCAO (**A**–**C** and **G**–**I**) and p-MCAO (**D**–**F** and **J**–**L**) at 1 d (**A**–**F**) and 7 d (**G**–**L**) post stroke. Ischemic changes indicating cell death characterized by nuclear pyknotic changes were observed within the ischemic areas (**B**,**E**,**H**,**K**). At 1 d post stroke, neutrophils were observed within ischemic areas after p-MCAO (**F**, arrows,**M**), and to a lesser extent after t-MCAO (**C**,**M**). At 7 d post stroke, more macrophages/microglia were observed within the ischemic areas after 90-min t-MCAO (**I**,**N**) compared to p-MCAO (**L**,**N**). The size of the ischemic areas was not significantly different between the groups at 1 d post stroke, but was significantly smaller 7 d after 90-min t-MCAO compared to p-MCAO (**O**). Results are representative of three replicates. Scale bars = 1 mm (**A**,**D**,**G**,**J**), 100 μm (**B**,**E**,**H**,**K**), and 50 μm (**C**,**F**,**I**,**L**). * *p* < 0.05 between stroke models (90-min t-MCAO vs. p-MCAO), within day (**M**–**O**) (*n* = 3, for each model). Abbreviations: H&E, hematoxylin and eosin; p-MCAO, permanent middle artery occlusion; t-MCAO, transient middle cerebral artery occlusion.

H&E staining within the ischemic areas (Figure 2A–F) revealed cell death characterized by nuclear pyknotic changes, which is thought to be attributed to the irreversible condensation of the chromatin and nucleus [28], after 90-min t-MCAO (Figure 2B) and p-MCAO (Figure 2E). Notably, accumulation of inflammatory cells, such as neutrophils, was more frequently observed in and around blood vessels after p-MCAO (Figure 2F,M) compared to 90-min t-MCAO (Figure 2C,M). We next compared brain histology at 7 d post stroke (Figure 2G–L). Although inflammatory cells morphologically identified as macrophages/microglia were observed within the ischemic areas following 90-min t-MCAO (Figure 2H,I) and p-MCAO (Figure 2K,L), significantly more macrophages/microglia were observed in mice after 90-min t-MCAO (Figure 2N).

We then evaluated the size of the ischemic areas. Although the sizes were not significantly different between the 90-min t-MCAO and p-MCAO groups at 1 d post stroke, the 90-min t-MCAO group showed significantly smaller ischemic areas compared to p-MCAO at 7 d post stroke (Figure 2O). These findings indicate that early reperfusion reduced the ischemic area size over time.

### 3.3. Early Reperfusion Promotes Accumulation of Anti-Inflammatory M2 Macrophage/Microglia Following Ischemic Stroke

**Figure 3:**
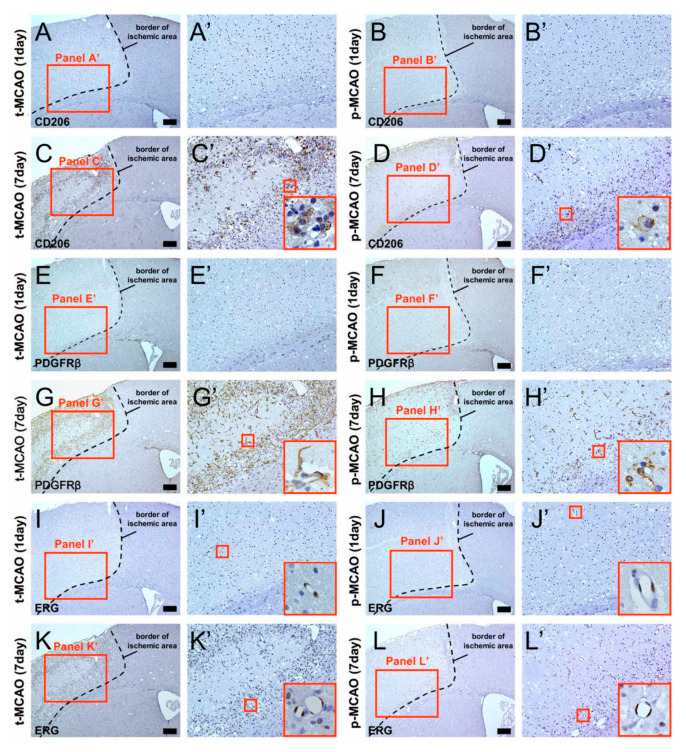

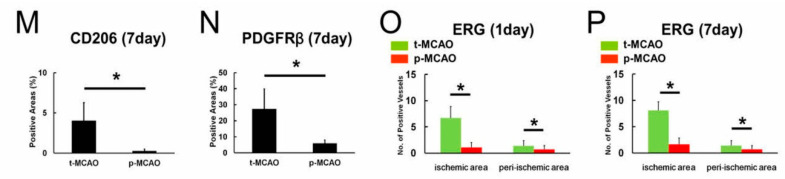
Immunohistochemistry for CD206, PDGFRβ, and ERG at 1 d and 7 d after 90-min t-MCAO or p-MCAO. CD206 (**A**,**A’**,**B**,**B’**) and PDGFRβ (**E**,**E’**,**F**,**F’**) were minimally expressed 1 d after 90-min t-MCAO (**A,A’,E,E’**) and p-MCAO (**B**,**B’**,**F**,**F’**). However, at 7 d post stroke, many CD206^+^ (**C**,**C’**,**D**,**D’**) and PDGFRβ^+^ cells (**G**,**G’**,**H**,**H’**) were observed within the ischemic areas after 90-min t-MCAO (**C**,**C’**,**G**,**G’**) and p-MCAO (**D**,**D’**,**H**,**H’**). Quantitative analysis at 7 d post stroke showed that CD206^+^ (**M**) and PDGFRβ^+^ areas (**N**) were significantly larger in mice after 90-min t-MCAO compared to p-MCAO. ERG^+^ endothelial cells were observed within the ischemic areas 1 d (**I**,**I’**,**J**,**J’**) and 7 d (**K**,**K’**,**L**,**L’**) after 90-min t-MCAO (**I**,**I’**,**K**,**K’**) and p-MCAO (**J**,**J’**,**L**,**L’**). However, quantitative analysis at 1 d (**O**) and 7 d (**P**) post stroke showed that the number of ERG^+^ vessels was significantly higher within the ischemic and peri-ischemic areas after 90-min t-MCAO compared to p-MCAO. Results are representative of three replicates. Scale bars = 200 µm (**A**–**L**). * *p* < 0.05 between stroke models (90-min t-MCAO vs. p-MCAO) (**M**–**P**) (*n* = 3, for each model). Abbreviations: CD206, cluster of differentiation 206; ERG, ETS-related gene; PDGFRβ, platelet derived growth factor receptor-beta; p-MCAO, permanent middle artery occlusion; t-MCAO, transient middle cerebral artery occlusion.

To investigate the mechanism by which early reperfusion may accelerate reductions in ischemic area size, we next investigated the expression pattern of CD206. This is a marker of anti-inflammatory M2 macrophage/microglia, which are known to be associated with tissue repair [29,30,31]. Immunohistochemistry 1 d post stroke revealed a few CD206^+^ cells within the ischemic areas produced by 90-min t-MCAO (Figure 3A,A’) and p-MCAO (Figure 3B,B’). Immunohistochemistry 7 d post stroke revealed an increase in CD206^+^ cells within the ischemic areas produced by 90-min t-MCAO (Figure 3C,C’) and p-MCAO (Figure 3D,D’). Quantitative analysis showed that, although the CD206^+^ regions within the ischemic areas were not significantly different at 1 d post stroke between treatment groups (90-min t-MCAO, 0.007 ± 0.022; p-MCAO, 0.018 ± 0.045), they were significantly higher at 7 d following 90-min t-MCAO compared to that following p-MCAO (Figure 3M). Notably, many CD206^+^ M2 macrophage/microglia were observed in not the central but the peripheral zones within the ischemic areas after 90-min t-MCAO (Figure 3C,C’).

### 3.4. Early Reperfusion Preserves and Promotes Healing Processes in Vascular Niches Following Ischemic Stroke

The neurovascular unit, which includes neural cells (neurons, astrocytes), endothelial cells, and pericytes, comprise the brain [32]. Although H&E staining 1 d post stroke showed that cells within the ischemic areas undergo cell death (Figure 2B,E), their precise cellular traits remain unclear. Immunohistochemistry showed that cells within the MCA lacked neuronal (MAP2^+^) and astrocyte (S100β^+^) markers after 90-min t-MCAO (MAP2^+^ cells, Supplementary Figure S1A–C; S100β^+^ cells, Supplementary Figure S1I–K) and p-MCAO (MAP2^+^ cells, Supplementary Figure S1E–G; S100β^+^ cells, Supplementary Figure S1M–O). However, MAP2^+^ neurons and S100β^+^ astrocytes were present in the ipsilateral non-ischemic MCA after 90-min t-MCAO (MAP2^+^ cells, Supplementary Figure S1A,B,D; S100β^+^ cells, Supplementary Figure S1I,J,L) and p-MCAO (MAP2^+^ cells, Supplementary Figure S1E,F,H; S100β^+^ cells, Supplementary Figure S1M,N,P). These findings confirm that mature neural cells, including neurons and glia, undergo cell death 1 d following both ischemic treatments.

Although these mature neural cells underwent cell death (Supplementary Figure S1), previous studies have shown that vascular cells, such as pericytes [11,12,13,14] and endothelial cells [9,10] were resistant to ischemia/hypoxia and could survive within ischemic areas even after stroke. Thus, we next examined whether pericytes (PDGFRβ^+^) and endothelial cells (ERG^+^) were present within the ischemic areas. Immunohistochemistry at 1 d post stroke showed PDGFRβ^+^ pericytes within the ischemic areas after 90-min t-MCAO (Figure 3E,E’) and p-MCAO (Figure 3F,F’). Similarly, immunohistochemistry 1 d post stroke showed ERG^+^ endothelial cells within the ischemic areas after 90-min t-MCAO (Figure 3I,I’) and p-MCAO (Figure 3J,J’). Quantitative analysis showed that PDGFRβ^+^ areas within the ischemic areas were not significantly different between the treatment groups (90-min t-MCAO, 0.180 ± 0.102; p-MCAO, 0.181 ± 0.127), but the number of ERG^+^ vessels within the peri-ischemic areas were significantly higher after 90-min t-MCAO compared to p-MCAO (Figure 3O). These findings indicate that the observed vascular cells are resistant to ischemic insult. Further, early reperfusion exerts a cytoprotective effect in endothelial cells, but not pericytes, during acute period following stroke.

Increasing evidence shows that pericytes and endothelial cells play an important role in wound healing following various CNS injuries, including ischemic stroke [10,33,34,35,36]. Thus, we next investigated PDGFRβ^+^ and ERG^+^ cell expression patterns 7 d post stroke. Immunohistochemistry revealed that PDGFRβ^+^ pericytes and ERG^+^ endothelial cells were present within the ischemic areas after 90-min t-MCAO (PDGFRβ^+^ cells: Figure 3G,G’; ERG^+^ cells; Figure 3K,K’) and p-MCAO (PDGFRβ^+^ cells; Figure 3H,H’; ERG^+^ cells; Figure 3L,L’). Quantitative analysis showed that PDGFRβ^+^ areas within the ischemic areas (Figure 3N) and the number of ERG^+^ vessels within the peri-ischemic areas (Figure 3P) were significantly higher following 90-min t-MCAO compared to p-MCAO. Similar to the CD206^+^ M2 macrophage/microglia localization, we observed many PDGFRβ^+^ pericytes and ERG^+^ endothelial cells near the ischemic area border after 90-min t-MCAO. These results suggest that early reperfusion promoted healing processes via M2 macrophage/microglia and vascular cells, predominantly at the peripheral zones of the ischemic areas during acute period following stroke.

### 3.5. Early Reperfusion Promotes NSPC Production Following Ischemic Stroke

**Figure 4:**
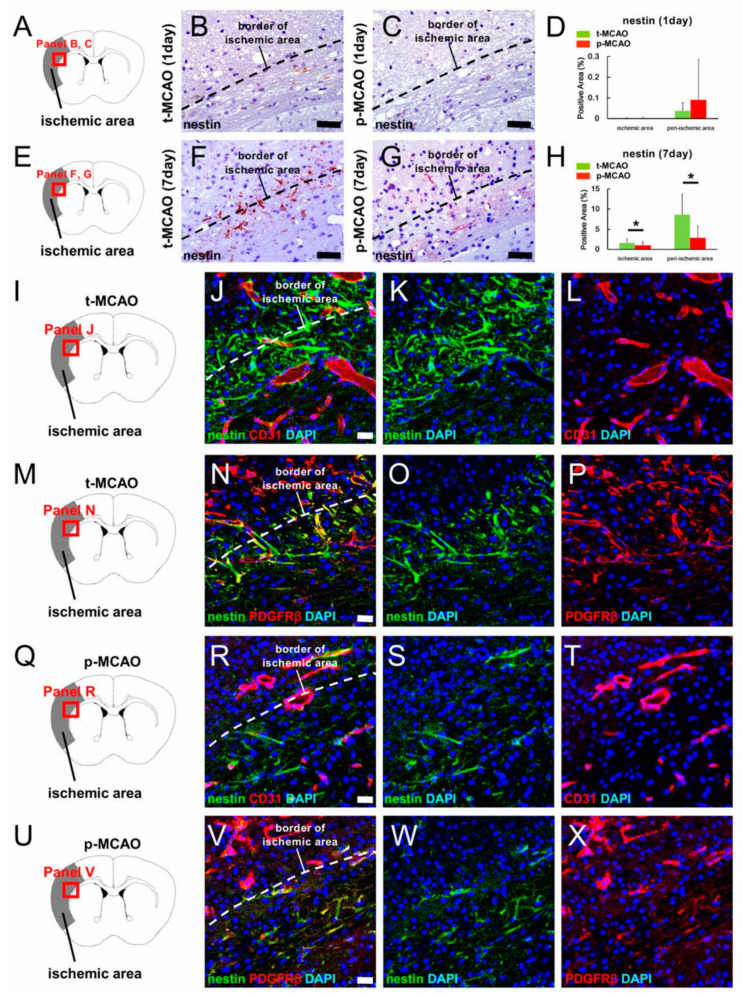
Immunohistochemistry for nestin at 1 d (**A**–**C**) and 7 d (**E**–**G**) after 90-min t-MCAO (**B**,**F**) or p-MCAO (**C**,**G**). At 1 d post stroke, nestin^+^ areas were not significantly different between the stroke models within the ischemic and peri-ischemic areas (**D**), but were significantly larger in both regions 7 d after 90-min t-MCAO compared to p-MCAO (**H**). Immunohistochemistry at 7 d after 90-min t-MCAO (**I**–**P**) or p-MCAO (**Q**–**X**) showed that some nestin^+^ cells within ischemic and peri-ischemic areas localized near CD31^+^ endothelial cells [90-min t-MCAO, nestin (**J** and **K**: green), CD31 (**J** and **L**: red), DAPI (**J**–**L**: blue); p-MCAO, nestin (**R** and **S**: green), CD31 (**R** and **T**: red), DAPI (**R**–**T**: blue)] and co-expressed PDGFRβ [90-min t-MCAO, nestin (**N** and **O**: green), PDGFRβ (**N** and **P**: red), DAPI (**N**–**P**: blue); p-MCAO, nestin (**V** and **W**: green), PDGFRβ (**V** and **X**: red), DAPI (**V**–**X**: blue)]. Results are representative of three replicates. Scale bars = 50 µm (**B**,**C**,**F**,**G**) and 20 µm (**J**,**N**,**R**,**V**). * *p* < 0.05 between stroke models (90-min t-MCAO vs. p-MCAO) (**H**) (*n* = 3, for each model). Abbreviations: DAPI, 4′,6-diamidino-2-phenylindole; PDGFRβ, platelet derived growth factor receptor-beta; p-MCAO, permanent middle artery occlusion; t-MCAO, transient middle cerebral artery occlusion.

We next investigated whether early reperfusion induced NSPCs by evaluating the number of cells expressing nestin (a marker for NSPCs) within the ischemic areas. One day post stroke, nestin^+^ cells were scarcely observed within and around the ischemic areas after 90-min t-MCAO (Figure 4A,B) and p-MCAO (Figure 4A,C). However, 7 d post stroke, many nestin^+^ cells were observed within and around the ischemic areas after 90-min t-MCAO (Figure 4E,F) and p-MCAO (Figure 4E,G). In contrast, nestin^+^ cells were scarcely observed in the contralateral non-ischemic MCA after 90-min t-MCAO and p-MCAO (data not shown). Quantitative analysis showed that nestin^+^ areas within the ischemic and peri-ischemic areas were not significantly different between the models at 1 d post stroke (Figure 4D), but were significantly larger at 7 d after 90-min t-MCAO compared to p-MCAO (Figure 4H).

These results indicate that early reperfusion promoted nestin^+^ cell production. Ischemia induces the production of nestin^+^ NSPCs partially via reactive pericytes [12,13,14]. Therefore, we further examined nestin^+^ cell traits within the ischemic areas. Immunohistochemistry at 7 d post stroke confirmed that nestin^+^ cells were observed within and around ischemic areas after 90-min t-MCAO (Figure 4I–P) and p-MCAO (Figure 4Q–X). In addition, nestin^+^ cells were largely localized near CD31^+^ blood vessels in both models (Figure 4I–L,Q–T), and some co-expressed PDGFRβ (Figure 4M–P,U–X). These results support previous studies showing that nestin^+^ NSPCs induced following ischemic stroke are partially derived from brain pericytes [12,13,14].

**Figure 5:**
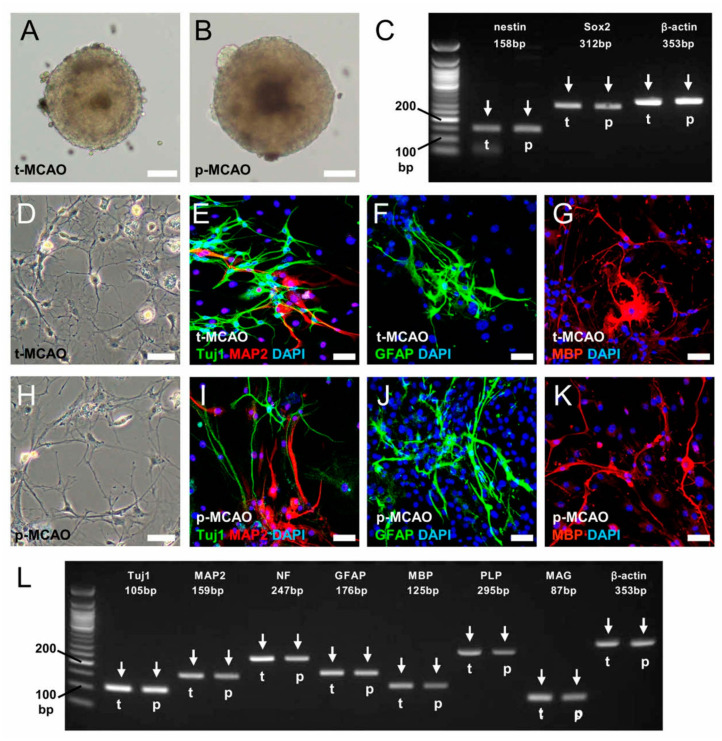
Following incubation of ischemic areas, neurosphere-like cell clusters were obtained from mice after 90-min t-MCAO (**A**) and p-MCAO (**B**). RT-PCR was used to analyze the cell clusters for NSPC markers (t-MCAO, t, arrows; p-MCAO, p, arrows), including nestin and Sox2 (**C**). After differentiation, immunohistochemistry showed that these cells (90-min t-MCAO (**D**), p-MCAO (**H**)) displayed the neuronal markers Tuj1 (90-min t-MCAO (**E**: green), p-MCAO (**I**: green)) and MAP2 (90-min t-MCAO (**E**: red), p-MCAO (**I**: red)), the astrocytic marker GFAP (90-min t-MCAO (**F**: green), p-MCAO (**J**: green)), and oligodendrocyte marker MBP (90-min t-MCAO (**G**: red), p-MCAO (**K**: red)). Nuclei were counterstained with DAPI (**E**–**G**, **I**–**K**: blue). RT-PCR analysis revealed that the differentiated cells displayed various neural markers, including for neurons (Tuj1, MAP2, NF), astrocytes (GFAP), and oligodendrocytes (MBP, PLP, MAG) (**L**). Results are representative of three replicates. Scale bars = 100 µm (**A**,**B**) and 50 µm (**D**–**K**). Abbreviations: DAPI, 4’,6-diamidino-2-phenylindole; GFAP, glial fibrillary acidic protein; MAG, myelin-associated glycoprotein; MAP2, microtubule-associated protein 2; MBP, myelin basic protein; NF, neurofilament; PLP, proteolipid protein; p-MCAO, permanent middle artery occlusion; t-MCAO, transient middle cerebral artery occlusion.

To further investigate nestin^+^ cell traits, we isolated cells from the ischemic areas following 90-min t-MCAO and p-MCAO and incubated them under conditions that promote the formation of neurosphere-like cell clusters [20] (Figure 5A,B). RT-PCR analysis showed that the neurosphere-like cell clusters obtained from both models exhibited NSPC markers, including nestin and Sox2 (Figure 5C). In addition, immunohistochemistry showed that differentiated cells (Figure 5D,H) expressed the neuronal markers Tuj1 and MAP2 (Figure 5E,I), the astrocytic marker GFAP (Figure 5F,J), and the oligodendrocyte marker MBP (Figure 5G,K). RT-PCR analysis also showed that differentiated cells expressed various neural markers, including neurons (Tuj1, MAP2, neurofilament), astrocytes (GFAP), and oligodendrocytes (MBP, PLP, MAG) (Figure 5L). These results confirm that NSPCs are induced after 90-min t-MCAO and p-MCAO.

### 3.6. Early Reperfusion Promotes Neuro-Vasculogenesis Following Ischemic Stroke

**Figure 6:**
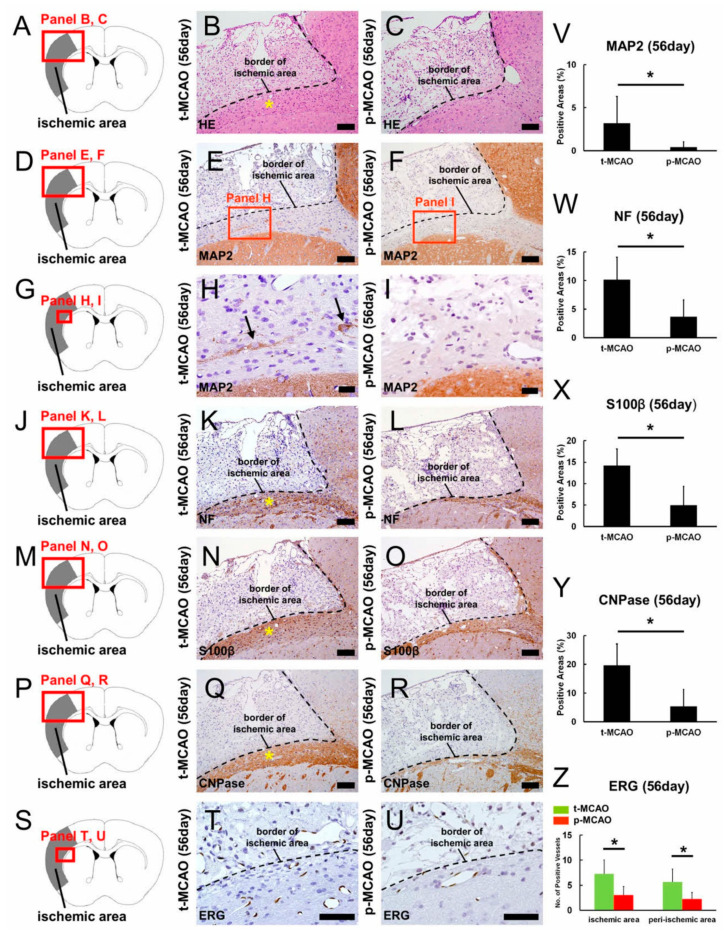
H&E staining (**A**–**C**) obtained 56 d after 90-min t-MCAO (**B**) and p-MCAO (**C**). The tissues around the ischemic area border were thicker after 90-min t-MCAO compared to p-MCAO (**B**, asterisk). Immunohistochemistry for MAP2 (**D**–**I**), neurofilament (**J**–**L**), S100β (**M**–**O**), and CNPase (**P**–**R**) at 56 d post stroke revealed that these neural markers were more prominent after 90-min t-MCAO (MAP2^+^ neurons (**E**,**H**, arrows), NF^+^ neurons (**K**, asterisk), S100β^+^ astrocytes (**N**, asterisk), CNPase^+^ oligodendrocytes (**Q**, asterisk)) compared to p-MCAO (MAP2^+^ neurons (**F**,**I**), NF^+^ neurons (**L**), S100β^+^ astrocytes (**O**), CNPase^+^ oligodendrocytes (**R**)). Immunohistochemistry at 56 d post stroke showed that more ERG^+^ endothelial cells (**S**–**U**) were observed after 90-min t-MCAO (**T**) compared to p-MCAO (**U**). Quantitative analysis showed that peri-ischemic areas positive for MAP2 (**V**), neurofilament (**W**), S100β (**X**), and CNPase (**Y**) markers were significantly larger after 90-min t-MCAO compared to p-MCAO. Quantitative analysis showed that the number of ERG^+^ vessels within and peri-ischemic areas was significantly greater after 90-min t-MCAO compared to p-MCAO (**Z**). Results are representative of three replicates. Scale bars = 100 μm (**B**,**C**,**E**,**F**,**K**,**L**,**N**,**O**,**Q**,**R**), 20 μm (**H**,**I**), and 50 μm (**T**,**U**). * *p* < 0.05 between stroke models (90-min t-MCAO vs. p-MCAO) (**V**–**Z**) (*n* = 3, for each model). Abbreviations: CNPase, 2’,3’-Cyclic-nucleotide 3’-phosphodiesterase; ERG, ETS-related gene; GFAP, glial fibrillary acidic protein; H&E, hematoxylin and eosin; MAP2, microtubule-associated protein 2; NF, neurofilament; p-MCAO, permanent middle artery occlusion; t-MCAO, transient middle cerebral artery occlusion.

We next investigated the effect of early reperfusion on CNS repair over extended periods of recovery. H&E staining at 56 d post stroke revealed thickened tissues around the border of the ischemic areas after 90-min t-MCAO (Figure 6A,B) compared to p-MCAO (Figure 6A,C), suggesting that reparative processes were facilitated after early reperfusion. As detailed in Section 3.5, early reperfusion increased the number of nestin^+^ NSPCs at peri-ischemic areas at 7 d post stroke (Figure 4H). In addition, we have previously shown that nestin^+^ NSPCs activated after ischemic stroke could differentiate into neurons and glia at peri-ischemic areas, but not within ischemic areas [10,37,38]. Based on these findings, we evaluated the peri-ischemic areas positive for neurons (MAP2^+^, neurofilament^+^), astrocytes (S100β^+^), and oligodendrocytes (CNPase^+^). One day post stroke, MAP2^+^ cells were scarcely observed after 90-min t-MCAO and p-MCAO (Supplementary Figure S2A–C), but were present at 56 d post stroke in both models (Figure 6D–I). Quantitative analysis showed that MAP2^+^ areas were not significantly different between the models at 1 d post stroke (Supplementary Figure S2D), suggesting that early reperfusion did not rescue neurons in peri-ischemic areas. However, MAP2^+^ areas were significantly larger at 56 d after 90-min t-MCAO compared to p-MCAO (Figure 6V). Similarly, neurofilament^+^ areas were significantly larger at 56 d after 90-min t-MCAO (Figure 6J,K,W) compared to p-MCAO (Figure 6J,L,W). These results indicate that, although neurogenesis occurred in the peri-ischemic areas in both models, early reperfusion promoted neurogenesis for a longer duration after t-MCAO.

We further investigated whether early reperfusion affected gliosis following ischemia. One day post stroke, only a few S100β^+^ cells were observed in peri-ischemic areas after 90-min t-MCAO and p-MCAO (Supplementary Figure S2E–G), but an abundance were observed at 56 d post stroke in both models (Figure 6M–O). Quantitative analysis showed that the number of S100β^+^ cells was not significantly different between the models at 1 d post stroke (Supplementary Figure S2H), suggesting that early reperfusion did not rescue astrocytes in peri-ischemic areas. However, S100β^+^ areas were significantly larger at 56 d after 90-min t-MCAO compared to p-MCAO (Figure 6X). Similarly, CNPase^+^ areas were significantly larger at 56 d after 90-min t-MCAO compared to p-MCAO (Figure 6P,Q,R,Y). These results indicate that, although gliosis occurred in the peri-ischemic areas in both models, early reperfusion promoted gliosis for a longer duration after t-MCAO.

We further investigated whether early reperfusion promoted vasculogenesis over extended periods of recovery. At 56 d post stroke, numerous ERG^+^ cells were observed in the peri-ischemic areas after 90-min t-MCAO and p-MCAO (Figure 6S–U). Quantitative analysis showed that the number of ERG^+^ vessels was significantly greater within and around the ischemic areas after 90-min t-MCAO compared to p-MCAO (Figure 6Z). These results indicate that early reperfusion promoted acute (Figure 3N–P) and long-term vasculogenesis after t-MCAO.

### 3.7. Early Reperfusion Prevents Neurological Behavioral Dysfunction Following Ischemic Stroke

We next investigated whether early reperfusion affected post-stroke neurological functions using a number of behavioral tests.

**Figure 7:**
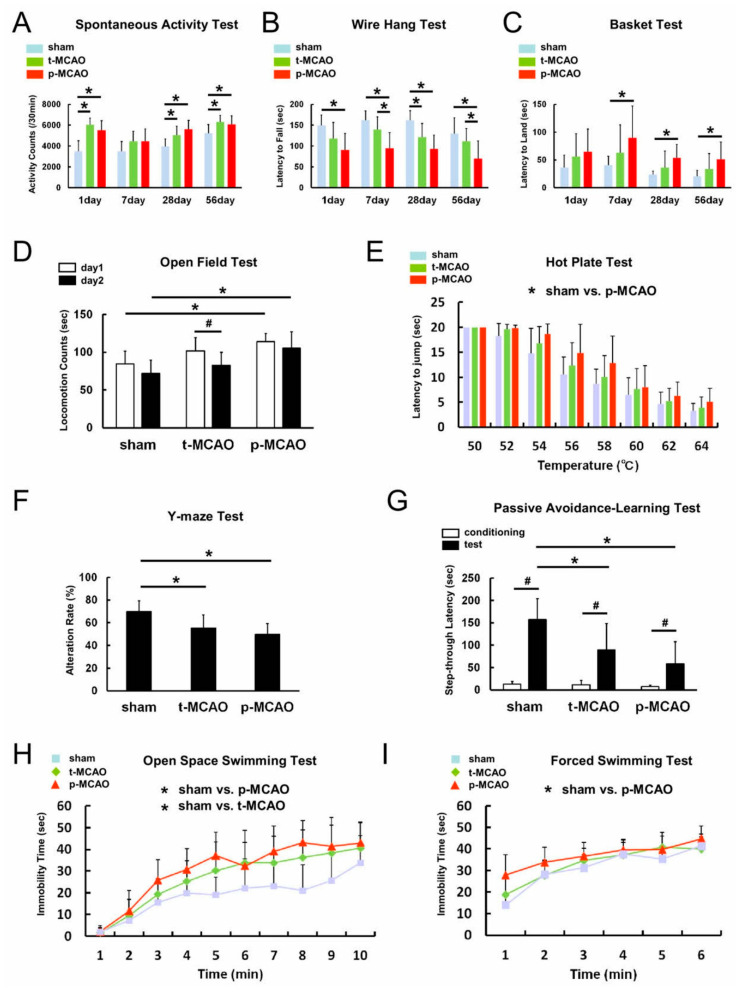
The spontaneous activity (**A**), wire hang (**B**), and basket (**C**) tests were performed 1, 7, 28, and 56 d post stroke. The open-field (**D**), hot plate (**E**), Y-maze (**F**), passive avoidance learning (**G**), open space swimming (**H**), and forced swimming (**I**) tests were performed 56 d post stroke. * *p* < 0.05 between groups (sham-operation vs 90-min t-MCAO, sham-operation vs. p-MCAO, or 90-min t-MCAO vs. p-MCAO), within day (**A**–**I**). ^#^ *p* < 0.05 between days 1 and 2 (**D**). ^#^ *p* < 0.05 between conditioning and test days (**G**). (*n* = 12 for sham, *n* = 16 for t-MCAO, *n* = 11 for p-MCAO). Abbreviations: p-MCAO, permanent middle artery occlusion; t-MCAO, transient middle cerebral artery occlusion.

The spontaneous activity test revealed significantly greater activity after 90-min t-MCAO and p-MCAO compared with the sham-operation at 1 d post stroke, with no significant difference between the 90-min t-MCAO and p-MCAO models (Figure 7A). Similar results were observed at 28 and 56 d post stroke (Figure 7A). These results indicate that, compared with sham-operated mice, the stroke models induced similar degrees of aberrant activity at the acute phase that persisted for an extended period of time.

The wire hang test revealed significantly reduced hang time after p-MCAO, but not 90-min t-MCAO, compared to the sham-operation at 1 d post stroke (Figure 7B), indicating that early reperfusion prevented neurological deficits in this motor task. In addition, hang time was significantly greater at 7 and 56 d after 90-min t-MCAO compared to p-MCAO. These results suggest that early reperfusion improved motor function at later time points.

The basket test showed that there was no significant difference between the groups in climbing behavior at 1 d post stroke. However, climbing behavior was significantly delayed at 7, 28, and 56 d after p-MCAO, but not 90-min t-MCAO, compared to the sham-operation (Figure 7C). Combined with the wire hang test findings, these data indicate that early reperfusion prevented losses in motor function that developed over time in the p-MCAO group.

Further, using multitasking behavioral tests, including the open-field test, hot plate test, Y-maze test, passive avoidance-learning test, open space swimming test, and forced swimming test, we investigated the effect of early reperfusion over extended recovery in detail.

The open-field test was performed to evaluate spontaneous locomotive activity using hyperactivity and habituation indices. Locomotive activity was assessed for two consecutive days within the groups. Activity significantly decreased on the second day only within the 90-min t-MCAO group. Comparing activity between groups on day 1 and 2 revealed that locomotive activity was significantly increased after p-MCAO, but not 90-min t-MCAO, compared to the sham operation at 1 and 2 d post stroke (Figure 7D). These results suggest that early reperfusion suppressed hyperactivity induced by lethal ischemia.

The hot plate test was performed to evaluate sensitivity to thermal nociception. The results showed that the latency to jump was significantly increased after p-MCAO, but not 90-min t-MCAO, compared to the sham operation (Figure 7E).

The Y-maze test was performed to evaluate spatial working memory measured by the frequency to alternate directions. The results showed that the 90-min t-MCAO and p-MCAO groups showed similar alternation rates that were significantly decreased compared to the sham-operation group (Figure 7F). These results suggest that dysfunctional spatial working memory following ischemic stroke is present over an extended period of time in both models.

The passive avoidance-learning test was performed to evaluate long-term memory function by measuring the latency to enter a chamber where the mouse was previously exposed to an electrical shock. The passive latency to enter the shock-paired chamber was significantly increased in all groups compared to their baseline latency. However, 90-min t-MCAO and p-MCAO significantly reduced the latency to enter the shock-paired chamber on the test day compared to the sham-operation group, indicating reduced passive avoidance behavior (Figure 7G). These results suggest that long-term memory dysfunction following ischemic stroke is present over an extended period of time in both models.

The open space swimming test was performed to evaluate depression-like symptoms following ischemic stroke by observing the time spent immobile. Immobility time was significantly greater after 90-min t-MCAO and p-MCAO compared to the sham operation (Figure 7H). These results indicate that similar degrees of depression-like symptoms were observed over an extended period of time in both models.

The forced swim test was performed to further investigate the degree of depression-like symptoms following ischemic stroke in a smaller water-filled arena. In this task, the immobility time was significantly higher after p-MCAO, but not t-MCAO, compared to the sham-operation (Figure 7I). In opposition to the open space swim test, these results suggest that early reperfusion suppressed depression-like symptoms induced by lethal ischemia over an extended period of time.

### 3.8. Early Reperfusion Does Not Exhibit Negative Complication Compared with Permanent Ischemia

**Figure 8:**
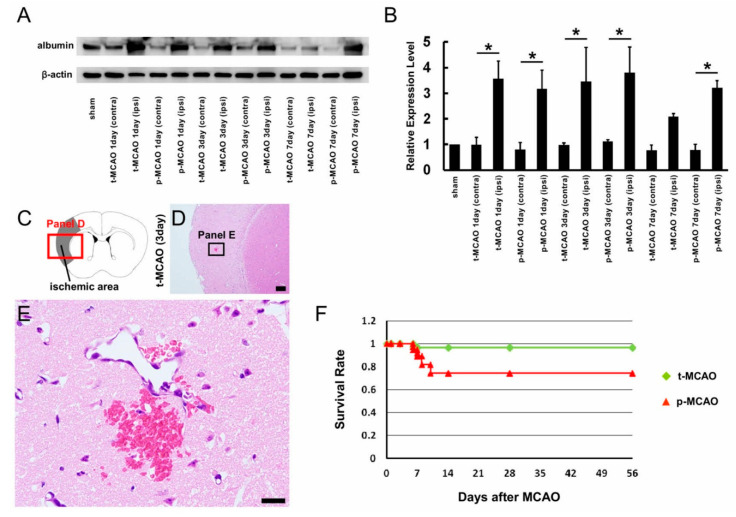
Western blot analysis of albumin expression in brain samples obtained from ipsilateral ischemic or contralateral non ischemic MCA areas after 90-min t-MCAO and p-MCAO (**A**,**B**). At 1 and 3 d post stroke, albumin expression levels in the ipsilateral ischemic MCA areas were significantly greater than in the contralateral non-ischemic MCA areas after 90-min t-MCAO and p-MCAO (**B**). At 7 d post stroke, albumin expression levels in the ipsilateral ischemic MCA areas were significantly greater than in the contralateral non-ischemic MCA areas after p-MCAO, but not 90-min t-MCAO (**B**). H&E staining was performed for mice after 90-min t-MCAO and p-MCAO. H&E staining indicated hemorrhagic transformation after 90-min t-MCAO at 3 d post stroke. (*n* = 18, for each model) (**C**–**E**). A Kaplan–Meier curve showed that survival rates were not significantly different between the stroke models (*n* = 49 for t-MCAO, *n* = 56 for p-MCAO) (**F**). * *p* < 0.05 between brain regions (**B**) (*n* = 3, for each sample). Scale bars = 200 µm (**D**) and 20 µm (**E**). Abbreviations: contra, contralateral; H&E, hematoxylin and eosin; ipsi, ipsilateral; p-MCAO, permanent middle artery occlusion; t-MCAO, transient middle cerebral artery occlusion; MCA, middle cerebral artery.

Finally, we investigated whether early reperfusion affected the incidence of complications, including increased BBB permeability, hemorrhagic transformation, and increased mortality. BBB permeability was investigated by quantifying albumin expression levels using western blot analysis after 90-min t-MCAO and p-MCAO (Figure 8A). Albumin expression in the ischemic areas was significantly greater than in the contralateral non-ischemic MCA areas at 1 and 3 d after 90-min t-MCAO and p-MCAO (Figure 8B). Albumin expression within the ischemic areas was not significantly different between stroke models at any timepoint (1, 3, and 7 d post stroke; Figure 8B). At 7 d post stroke, albumin expression remained elevated in the ischemic areas compared to the contralateral non-ischemic MCA areas after p-MCAO, but not 90-min t-MCAO (Figure 8B). These findings indicate that early reperfusion did not increase BBB permeability, but rather tended to decrease it at 7 d post stroke.

We next evaluated the frequency of hemorrhagic transformation between the two groups using H&E staining at 1, 3, 7, 14, 28, and 56 d post stroke. Local hemorrhagic transformation was observed in only one mouse after 90-min t-MCAO (Figure 8C–E) (1 d, 0/3 mice; 3 d, 1/3 mice; 7 d, 0/3 mice; 14 d, 0/3 mice; 28 d, 0/3 mice; 56 d, 0/3 mice), whereas it was observed in two mice after p-MCAO (data not shown; 1 d, 0/3 mice; 3 d, 1/3 mice; 7 d, 1/3 mice; 14 d, 0/3 mice; 28 d, 0/3 mice; 56 d, 0/3 mice). The total overall incidence of hemorrhagic transformation was not significantly different between the 90-min t-MCAO and p-MCAO groups (*p* = 0.54). These results indicate that early reperfusion did not alter the rates of hemorrhagic transformation.

Finally, we compared the survival rates of the mice used in these experiments. Eight weeks post stroke, 6/56 mice died in the p-MCAO groups (survival rate, 74.3%), whereas 1/49 mice died in the 90-min t-MCAO groups (survival rate, 96.6%). These results were compared using a Kaplan–Meier curve (Figure 8F). Notably, a logrank test showed that survival rates were not significantly different between the stroke models (*p* = 0.10), indicating that early reperfusion did not increase the mortality rate following ischemic stroke.

## 4. Discussion

This study demonstrated for the first time that early reperfusion provides a beneficial effect on behavioral outcomes indicative of neurological function, even when brains were subjected to lethal ischemia causing mature neural cell death. In addition, early reperfusion does not increase negative complications, including hemorrhagic transformation and mortality. These results indicate that early reperfusion can be an intervention therapy option for patients with lethal ischemia.

The current study found that neurological deficits were attenuated at 1 d after 90-min t-MCAO compared to p-MCAO. The therapeutic effects of reperfusion are primarily attributed to neuronal cytoprotection at the penumbra regions, where cells still survive despite decreased blood flow [39]. A previous study using neuroimaging showed that penumbra regions were detectable within the ischemic and peri-ischemic areas [40]. Although the precise penumbra regions were not identified in the current study, MAP2^+^ neurons were not observed within the ischemic areas at 1 d post stroke in either model. Further, the number of MAP2^+^ neurons in the peri-ischemic areas at 1 d post stroke were not significantly different between the models. These results strongly suggest that early reperfusion following 90-min t-MCAO did not rescue neurons in the ischemic areas, including the penumbra. However, the ability of earlier reperfusion, such as at the 20-min time point, to rescue neuronal death in the penumbra cannot be ruled out by the current studies.

Similar to the MAP2^+^ findings, early reperfusion following 90-min t-MCAO did not affect S100β^+^ astrocyte survival at 1 d post stroke. However, we found that vascular cells, like endothelial cells and pericytes, survived at 1 d post stroke even within the ischemic areas. In addition, early reperfusion increased the number of endothelial cells that survived. These results indicate that early reperfusion following 90-min t-MCAO did not rescue mature neural cells, but did rescue vascular cells during the acute phase following ischemia. This also suggests that early perfusion’s ability to preserve blood vessels may contribute to the reduced neurological deficits observed 1 d after 90-min t-MCAO. The precise mechanism underlying blood vessel conservation in this model remains unclear. However, we found that neutrophils were increased in and around blood vessels after p-MCAO compared to 90-min t-MCAO. However, it is unclear why larger numbers of neutrophils were accumulated in the ischemic areas after p-MCAO. Activated neutrophils following ischemic stroke are associated with brain damage, including compromised blood vessels [41]. Therefore, it is possible that neutrophils facilitated endothelial cell injury within ischemic areas, thereby decreasing the number of ERG^+^ endothelial cells after p-MCAO.

The current study found that early reperfusion reduced the size of the ischemic areas. Although the mechanism of this effect was not directly identified, we found that early reperfusion increased the number of anti-inflammatory CD206^+^ M2 macrophage/microglia, which are associated with tissue repair [29,30,31]. The reason for the presence of large numbers of CD206^+^ M2 macrophage/microglia within the ischemic areas after t-MCAO compared to that after p-MCAO remains unclear. However, it has been reported that macrophage/microglia originate in part from circulating monocytes and bone marrow-derived hematopoietic cells [42,43,44]. Thus, it is possible that larger numbers of these cells were accumulated in and around the ischemic areas following reperfusion. Alternatively, it is possible that reperfusion activated tissue-resident macrophage/microglia that were originally residing in the brain [45,46]. In addition, we found that early reperfusion increased the number of PDGFRβ^+^ pericytes, which are associated with wound healing following brain injuries [10,33,34,35]. Interestingly, we found that both CD206^+^ M2 macrophage/microglia and PDGFRβ^+^ pericytes were increased predominately in the peripheral zones of the ischemic areas. Although the precise relations between macrophage/microglia and pericytes in the brain following ischemia remain unclear, recent studies showed that macrophage/microglia originate, in part, from brain pericytes [18,47,48]. Conversely, a subset of pericytes are macrophage/microglia derivatives [49,50]. Thus, it is possible that macrophage/microglia and pericytes cooperatively contribute to CNS tissue repair.

Further, we found that early reperfusion promoted NSPC production. In vitro, neurosphere-like cell clusters isolated from mice after t-MCAO and p-MCAO differentiated into various lineages, including neurons, astrocytes, and oligodendrocytes. Previous studies have shown that various cell types, such as NSPCs in the subventricular zone (SVZ) [51], ependymal cells [52], reactive astrocytes [53,54], and oligodendrocyte precursor cells [55], are NSPC candidates. It has been well documented that SVZ-derived NSPCs migrate toward ischemic areas following ischemia [56]. However, increasing evidence shows that their migratory capacities are limited [38,57] and regionally activated stem cells likely function as NSPCs following ischemic stroke [12,13,20,21,22]. Although the precise origin of NSPCs following ischemic stroke remains unclear, the current results demonstrate that NSPCs within ischemic areas are at least partially derived from local pericytes [12,13,14]. Our finding that nestin^+^ cells that developed within and around the ischemic areas largely expressed PDGFRβ also supports this interpretation of the data.

Consistent with the in vitro findings, mature neural cells, such as MAP2^+^ neurons, S100β^+^ astrocytes, and CNPase^+^ oligodendrocytes, were observed in the peri-ischemic areas in mice after t-MCAO and p-MCAO. In addition, we found that early reperfusion increased the areas that were positive for these markers. These results suggest that early reperfusion following stroke accelerated neural repair in vivo, including neurogenesis and gliosis. Although the precise mechanism of early reperfusion-induced healing remains unclear, we have previously demonstrated that endothelial cells in the ischemic areas promoted NSPC proliferation and accelerated neurogenesis following ischemic stroke [10,37] by serving as stem cell niches. The current study further supports these findings by showing that nestin^+^ cells largely developed near vascular cells. Thus, it is possible that endothelial cells preserved by early reperfusion function as stem cell niches and promote neural repair, including neurogenesis and gliosis, during extended recovery.

In addition to neural regeneration, early reperfusion promoted PDGFRβ^+^ pericyte proliferation at acute period (7 d post stroke). Further, early reperfusion increased the number of ERG^+^ endothelial cells during acute (1 and 7 d post stroke) and chronic periods (56 d post stroke). These results show that early reperfusion exerted an acute protective effect on endothelial cells and promoted healing processes in vascular lineage cells at later time points. Functionally, we found that early reperfusion prevented behavioral expression of neurological damage at multiple time points. Although the precise mechanism remains unclear, it has been reported that not only neurons [58] but also glia [59] and vascular cells [10] contribute to brain function. Thus, the reduced behavioral symptoms of neurological loss over varying periods of time after t-MCAO in mice may be attributed, in part, to accelerated neural and vascular repair induced by early reperfusion.

Previous studies have shown that reperfusion can result in negative brain effects, including hemorrhagic transformation [60,61] and increased BBB permeability [62,63]. However, the present study found that early reperfusion did not increase the incidence of hemorrhagic transformation and mortality. In addition, we found that BBB permeability within the ischemic areas was not significantly different between the models, and was actually reduced at 7 d after 90-min t-MCAO compared to p-MCAO. Although the mechanism remains unclear, we found that endothelial cells and pericytes were increased in mice after 90-min t-MCAO compared to p-MCAO. Thus, increased vascular cells following early reperfusion may contribute to BBB reconstruction, thereby decreasing BBB permeability. Another possibility is that vascular cells promoted regeneration in their role as stem cell niches, as discussed above. These results indicate that early reperfusion following ischemia provided beneficial effects without increasing negative complications.

In this study, lethal ischemia was confirmed by TTC staining and histological findings. Unfortunately, we could not perform brain imaging, such as MRI, to detect lethal ischemia. However, a previous study showed that ischemic areas identified using TTC staining were reliably associated with high-intensity areas identified using T2-weighted MRI (T2WI) [40]. Considering this overlap, the current study suggests that early reperfusion may be useful for stroke patients who eventually revealed the findings of lethal ischemia (high-intensity areas) by T2-weighted MRI. The current study found that a short ischemic duration [60-min t-MCAO or more (90-min t-MCAO)] was able to induce mature neural cell death. The vulnerability of neurons and glia to ischemic insult under oxygen–glucose deprivation, which can mimic ischemia/hypoxia, has also been demonstrated by an in vitro study [64,65,66,67]. However, we found that the vascular cells survived within the ischemic areas even after p-MCAO. Thus, reperfusion therapy may enhance the healing process via vascular cells beyond previously proposed time points. However, the effects of early reperfusion might vary depending on time until reperfusion after MCAO. Thus, it is necessary to examine whether beneficial effects observed in mice after 90-min t-MCAO are also obtained following longer ischemic durations.

Our study has several limitations. For example, we used S100β, PDGFRβ, and ERG as markers for astrocytes, pericytes, and endothelial cells, respectively. However, previous studies have shown that these markers are also expressed in other cell types (e.g., S100β expression in subpopulation of neurons [68]; PDGFRβ expression in radial glia-like cells [69]; and ERG expression in early myeloid cells [70]). In addition, a previous study has shown that cells localized outside the brain (e.g., bone-marrow cells) transdifferentiate into vascular cells, including pericytes and endothelial cells [71]. Thus, the presence of vascular cells within the ischemic areas might partly be attributed to the cells that migrate into the brain after the ischemic stroke.

In conclusion, we showed that early reperfusion reduced blood vessel damage, accelerated neuroregenerative and vaculogenerative reparative processes, and reduced behavioral expression of neurological damage. The precise mechanisms underlying these events should be clarified in future studies. However, the current study showed that early reperfusion might be a useful treatment in stroke patients that experienced lethal brain ischemia characterized by death of mature neurons and glia.

## Figures and Tables

**Table 1:** List and sequence of mouse primers used for RT-PCR analysis.

Primers	Sequence (5′-3′) (F: forward; R: reverse)	Size
β-actin	F: GCTCGTCGTCGACAAGGGCTC; R: CAAACATGATCTGGGTCATCTTCTC	353 bp
GFAP	F: TCGGCCAGTTACCAGGAGG; R: ATGGTGATGCGGTTTTCTTCG	176 bp
MAG	F: CAAGTCCCGCACACAAGTG; R: AGCAGGGTACAGTTTCGTAGG	87 bp
MAP2	F: CTCATTCGCTGAGCCTTTAGAC; R: ACTGGAGGCAACTTTTCTCCT	159 bp
MBP	F: TCACAGCGATCCAAGTACCTG; R: CCCCTGTCACCGCTAAAGAA	125 bp
nestin	F: CGCTGGAACAGAGATTGGAAG; R: CATCTTGAGGTGTGCCAGTT	158 bp
NF	F: CCGTACTTTTCGACCTCCTACA; R: CTTGTGTGCGGATAGACTTGAG	247 bp
PLP	F: TGAGCGCAACGGTAACAGG; R: GGGAGAACACCATACATTCTGG	295 bp
Sox2	F: TTGGGAGGGGTGCAAAAAGA; R: CCTGCGAAGCGCCTAACGTA	312 bp
Tuj1	F: TGAGGCCTCCTCTCACAAGT; R: GGCCTGAATAGGTGTCCAAA	105 bp

## Supplementary Materials

The following are available online at https://www.mdpi.com/2073-4409/9/6/1374/s1, Figure S1: Immunohistochemistry for MAP2 and S100β 1 d after 90-min t-MCAO and p-MCAO (Part 1), Figure S2: Immunohistochemistry for MAP2 and S100β 1 d after 90-min t-MCAO and p-MCAO (Part 2).
