# Supplementary material for: Early Reperfusion Following Ischemic Stroke Provides Beneficial Effects, Even After Lethal Ischemia with Mature Neural Cell Death
Source: Cells. 2020 Jun 1;9(6):1374. doi: 10.3390/cells9061374 (PMC7349270; doi:10.3390/cells9061374)
Supplement: Supplementary file 1 [file cells-09-01374-s001.pdf]

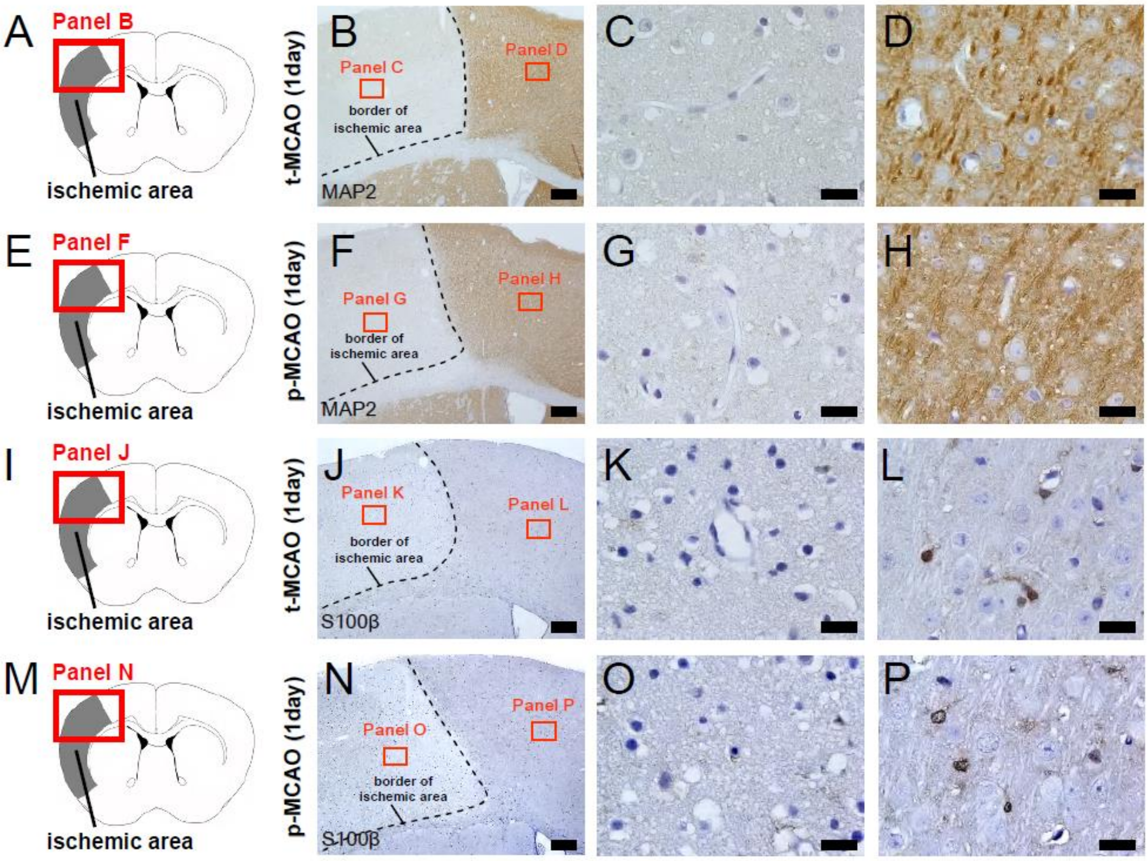

Supplementary Figure S1: Immunohistochemistry for MAP2 (A-H) and S100β (I-P) 1 d after 90-min t-MCAO (B-D, J-L) and p-MCAO (F-H, N-P). Immunohistochemistry showed that MAP2$^+$ cells were not observed within the ischemic areas after 90-min t-MCAO (B, C) and p-MCAO (F, G), but were observed in ipsilateral non-ischemic areas after 90-min t-MCAO (B, D) and p-MCAO (F, H). Immunohistochemistry showed that S100β$^+$ cells were not observed within the ischemic areas after 90-min t-MCAO (J, K) and p-MCAO (N, O), but were observed in the ipsilateral non-ischemic areas after 90-min t-MCAO (J, L) and p-MCAO (N, P). Results are representative of three replicates. Scale bars = 200 μm (B, F, J, N) and 20 μm (C, D, G, H, K, L, O, P). Abbreviations: contra, contralateral; ipsi, ipsilateral; MAP2, microtubule-associated protein 2; p-MCAO, permanent middle artery occlusion; t-MCAO, transient middle cerebral artery occlusion.

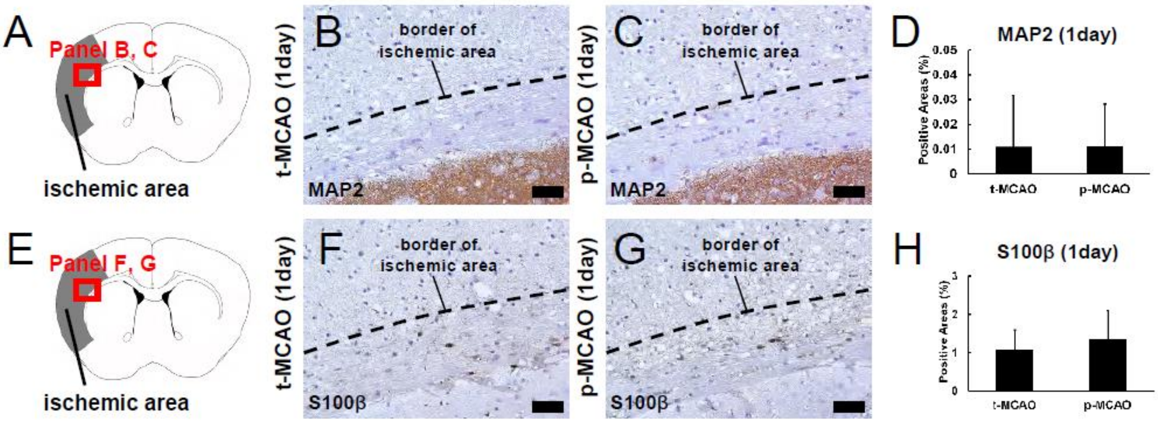

Supplementary Figure S2: Immunohistochemistry for MAP2 (A-D) and S100β (E-H) 1 d after 90-min t-MCAO (B, F) and p-MCAO (C, G). Immunohistochemistry showed that MAP2$^+$ cells were scarcely

observed at the peri-ischemic areas in mice after 90-min t-MCAO (B) and p-MCAO (C). Positive regions expressing MAP2 at the peri-ischemic areas were not significantly different between the stroke models (D). Immunohistochemistry showed that, although a few S100β$^+$ cells were observed at the peri-ischemic areas after 90-min t-MCAO (F) and p-MCAO (G), the positive regions expressing S100β at peri-ischemic areas were not significantly different between the stroke models (H). Results are representative of three replicates. Scale bars = 50 μm (B, C, F, G). Abbreviations: MAP2, microtubule-associated protein 2; p-MCAO, permanent middle artery occlusion; t-MCAO, transient middle cerebral artery occlusion.
